# Stress Distributions and Luminescent Responses of Mechanoluminescent Cylinders with Various Sizes and Loading Paths

**DOI:** 10.3390/ma18020331

**Published:** 2025-01-13

**Authors:** Chang-Ying Sun, Wei Liu, Xin Shi, Guang-Hui Rao, Jing-Tai Zhao

**Affiliations:** 1 School of Mechanical and Electrical Engineering, Guilin University of Electronic Technology, Guilin 541004, China; zyscy352016@163.com (C.-Y.S.); liuwei@mails.guet.edu.cn (W.L.); 2 Guangxi Key Laboratory of Information Materials, School of Materials Science and Engineering, Guilin University of Electronic Technology, Guilin 541004, China; shixin0324@163.com

**Keywords:** mechanoluminescent materials, size effect, loading path, stress distribution, luminescent response characteristics

## Abstract

Mechanoluminescent (ML) materials emit light by trapping and releasing charge carriers under mechanical stress. However, previous studies do not fully reveal the relationship between emitting light intensity and mechanical stress, thereby affecting the accuracy of stress measurement. This study addresses this gap by systematically investigating ML cylinders with various sizes and loading paths using theoretical analysis and simulations, focusing on the maximum contact stress, equivalent stress distribution, and the relationship between the strain energy density and light intensity at the point of maximum contact stress. In combination with experiments, the mechanical behavior and optical responses of ML cylinders under normal compressive forces reveal that the luminescence intensity is closely related to cylinder size and loading path, effectively reflecting stress distributions in objects of different sizes under complex stress conditions. Particularly, within the elastic range and under ideal conditions where lateral stress is ignored, the maximum contact stress is nearly equal to the equivalent stress. The equivalent stress is linearly related to the light intensity, while the strain energy density at the maximum contact stress point is proportional to the square root of the light intensity. This work promotes the application of ML materials in structural health monitoring and stress visualization.

## 1. Introduction

Mechanoluminescent (ML) materials, an emerging sensor candidate, emit light by trapping and releasing charge carriers under mechanical stress, exhibiting a high potential for structural health monitoring and dynamic stress visualization applications [1,2,3,4,5]. ML materials can possess high sensitivity and visual clarity, work without external power, and respond instantly to small stress changes, thus providing precise stress distribution images [6,7,8,9]. Typically, ML materials are embedded into an optical resin matrix similarly to skin-encased neurons. Each ML particle acts as a neuron, emitting light under stress and transmitting stress information [10,11,12,13,14,15]. This structure provides ML sensors with high sensitivity and resolution, enabling precise detection of stress distributions and changes. Therefore, ML materials offer an innovative solution for monitoring large structures such as bridges and buildings as well as for stress detection in flexible electronics and wearable devices with a broad application range [16,17,18,19,20,21,22].

Although many researchers have experimentally studied ML materials in cylindrical or thin-film structures with transparent polymer [2,14,23,24,25,26,27,28,29,30,31,32] to establish a relationship between load and luminescence during compression or stretching, the reported studies have hardly provided a consistent explanation for the dynamic states reflected by ML signals. Most studies have focused on relations between the ML intensity and the stress under specific dynamic conditions, such as constant pressure, constant tensile force, or constant strain rate. However, in practical applications, stress environments are often unforeseen with variable stresses and stress-change rates over time. This discrepancy limits the practical utility of ML materials in real-world dynamic monitoring applications, where stresses are neither uniform nor predictable.

The choice of cylindrical structures as the research object aligns with most existing literature [8,23,24,25,26,27,28,29,30], where ML cylinders are used to investigate their luminescent response under compression. Cylindrical structures exhibit well-characterized stress features that facilitate a deeper understanding of ML behavior under localized stress. The inherent symmetry of cylindrical structures also simplifies the analysis of stress distributions, making them ideal candidates for understanding the fundamental mechanics of ML behavior. For instance, in our previous work [24,25,26,27,28], we conducted systematic optimization of CaZnOS and SrZnSO-based cylinders, achieving multimodal luminescence and efficient red-light emission through the incorporation of Bi^3+^, Mn^2+^, and Nd^3+^ ions. We demonstrated significant enhancements in signal–noise ratio and saturation thresholds by regulating trap distributions, enabling superior dynamic response under high-pressure conditions. These materials were shown to hold great potential in anti-counterfeiting, stress-sensing, and dynamic monitoring applications. Additionally, Nd^3+^ doping effectively reduced afterglow interference and improved sensitivity to stress variations, offering a novel approach for high-precision stress monitoring. Similarly, Du et al. [29] examined LiNbO_3_:Pr^3+^ cylinders, revealing a strong correlation between luminescence intensity and stress distribution, and achieved efficient red luminescence by optimizing the Li^+^/Nb^+^ ratio. Zhang et al. [30] explored the ML characteristics of CaZnOS:Mn^2+^ cylinders, demonstrating a linear relationship between luminescence intensity and compression load, along with excellent reproducibility, underscoring its utility in stress visualization. However, despite these advances, the relationships between ML intensity, stress distribution, and dynamic loading conditions require further investigation, especially under variable mechanical conditions and across different cylinder geometries.

Most existing literature concludes that the luminescence intensity of ML cylinders is positively correlated with the applied load; however, the relationships between applied load, contact stress, internal stress, and luminescence intensity have not been thoroughly explored. Furthermore, the existing studies often assume a linear correlation between load and luminescence intensity, which oversimplifies the material’s response under complex mechanical conditions. Due to the structural complexity of ML cylinders, there is a nonlinear relationship between applied load and stress, making it insufficient to rely solely on the correlation between load and luminescence intensity to accurately reflect the stress state. Furthermore, the lack of research on the effect of ML cylinder size on maximum contact stress and internal equivalent stress distribution results in an unclear relationship between luminescence and stress, hindering the accurate interpretation of stress states from luminescence signals. In summary, relying solely on the relationship between applied load and luminescence data cannot effectively explain the visualization of the stressed object’s state. These limitations highlight the need for a comprehensive framework that accounts for the effects of stress distribution, geometric parameters, and dynamic loading paths on luminescence intensity.

In this work, we intend to fill the research gap by analyzing the mechanical response and the luminescence of ML cylinders under different mechanical conditions to achieve a better understanding of ML properties. By performing theoretical and simulation analyses of ML cylinders with various sizes, the maximum contact stress between the cylinder and the crosshead and the internal equivalent stress distribution have been derived. Compression tests were performed on cylinder samples with various sizes under different loading paths to measure the luminescence intensity and distribution at the maximum contact stress point. This study establishes the relationships among ML intensity, stress, cylinder size, and loading path, providing theoretical and experimental verification of these interdependencies. Furthermore, this study introduces a novel framework to correlate maximum strain energy density and its dynamic rate of change with ML properties, offering a deeper insight into the material’s response under variable mechanical conditions. This work uncovers the impact of the dynamic strain energy-change rate on the luminescent properties of ML cylinders, providing a novel understanding of their behavior under different mechanical conditions. The findings provide a theoretical basis and technical guidance for developing high-precision, high-sensitivity ML sensors.

## 2. Experimental and Simulation

### 2.1. Experimental

The fabrication process and luminescence measurements of ML cylinder samples are described as follows [31]. Bi-doped CaZnOS phosphors with the molar composition Ca(1–2*x*%)ZnOS%Bi^3+^, *x*%Li^+^ (*x* = 5) were synthesized from AR-grade CaCO_3_, ZnS, Bi_2_O_3_, and Li_2_CO_3_(all purchased from Aladdin Reagent Co., Ltd., Shanghai, China) via a high-temperature solid-state reaction. The raw materials were mixed and ground with ethanol in an agate mortar, then placed in an alumina crucible, and sintered at 1100 °C for 3 h under an argon flow in a horizontal tube furnace. After furnace cooling to 25 °C, the fabricated samples were ground into fine powders, mixed with optical-grade epoxy resin, vacuum-degassed, and molded into ML cylinders of various sizes. Compression tests were conducted in a dark room at room temperature using an AGS-X universal testing machine (Shimadzu Corporation, Kyoto, Japan). The applied loads ranged from 500 to 3000 N, and the crosshead speed varied from 1 to 10 mm/min. To ensure high accuracy in luminescence measurements, the fabricated ML cylinders were pre-exposed to 365 nm ultraviolet light for 5 min followed by a 60 min rest to stabilize the luminescent material. During compression, the luminescence intensity at the maximum contact stress region and its distribution on the surface were measured and recorded using a fiber-optic spectrometer (QE-Pro, Ocean Optics, Dunedin, FL, USA) and charge-coupled device camera (Baumer VLU-12, Frauenfeld, Switzerland). The luminescence response under an external force was subsequently analyzed through data processing.

### 2.2. Simulation Procedure

ANSYS Workbench developed by ANSYS, Inc. (Canonsburg, PA, USA), is an integrated simulation platform for studying structural mechanics, fluid dynamics, heat transfer, and electromagnetic properties, offering a unified interface from modeling to post-processing. A two-dimensional (2D) model of a cylinder compressed by crossheads was created using ANSYS Workbench with a cylinder radius of 10–15 mm and a crosshead dimension of 30 mm × 10 mm. Material properties were assigned as follows [33]: epoxy resin with an elastic modulus of 3780 MPa and Poisson’s ratio of 0.35, and stainless steel with an elastic modulus of 210 GPa and Poisson’s ratio of 0.3. Meshing was applied with a mesh size of 0.02 mm near the contact area and 0.2 mm elsewhere for accuracy. A 2D plane stress analysis was performed for a cylinder with a thickness ranging from 10 to 15 mm. Frictionless contact was assumed between the cylinder and crossheads, and a normal force load of 500–3000 N was applied. The selected path was a line segment connecting the maximum contact stress points between the cylinder and the upper and lower crossheads, passing through the center of the cylinder end face. This path line segment was divided into 199 segments to calculate the equivalent stress distribution along the path and calculate the maximum contact stress in the contact area.

## 3. Results and Discussion

### 3.1. ML Cylinder at a Constant Stress Rate

The crosshead applies a normal compression force to one end of an ML cylinder at a speed of 2 mm/min, with external forces ranging from 500 to 3000 N. The contact between the cylinder and crosshead is treated as a line between the cylinder and the flat surface of the crosshead. Because the cylinder remains within the elastic deformation range, the Hertzian contact theory [34,35,36,37] is used to calculate the contact stress under the following assumptions:

1. Elastic deformation: stress and strain are linearly related following Hooke’s Law.
2. Geometric approximation: the contact surface is small in comparison with the curvature radius, approximating a plane contact.
3. Load assumption: the load is perpendicular to the contact surface and the friction is ignored.

Based on these assumptions, the contact stress can be expressed by following formulae [38]:

(1) $$b=2\sqrt{\frac{F*r}{\pi *l{\;*\;E}^{*}}}$$

(2) $$\sigma \;(x)=\sigma_{max}\sqrt{1-{\left(\frac{x}{b}\right)}^{2}}$$

(3) $$\frac{1}{E^{*}}=\frac{1-{ʋ}_{1}^{2}}{E_{1}}+\frac{1-{ʋ}_{2}^{2}}{E_{2}}$$

(4) $$F=\int_{-b}^{b}\sigma \;(x)*l\;dx$$

where *F* is the normal force applied to the contacting bodies, and *r* is the effective radius of curvature, which is the radius of the cylinder for the crosshead–cylinder contact. E* is the effective elastic modulus, and *l* is the cylinder length. *E*_1_ and *ʋ*_1_ are the elastic modulus and Poisson’s ratio of the cylinder, while *E*_2_ and *ʋ*_2_ are those of the crosshead, respectively. *b* is the contact area half-width, and *σ* (*x*) is the contact stress in the region. From Equations (1)–(4), the following formula for the maximum contact stress between the cylinder and crosshead is derived:

(5) $$\sigma_{max}=\sqrt{\frac{F*E^{*}}{\pi *r*l}}$$

According to Johnson’s contact stress theory [38,39], the normal deformation between a plane and cylinder is represented by δ, which is calculated approximately as follows:

(6) $$\delta =\frac{F}{\pi *l}\cdot \frac{1-{ʋ}_{1}^{2}}{E_{1}}\cdot (2ln\frac{4r}{b}-1)$$

**Figure 1:**
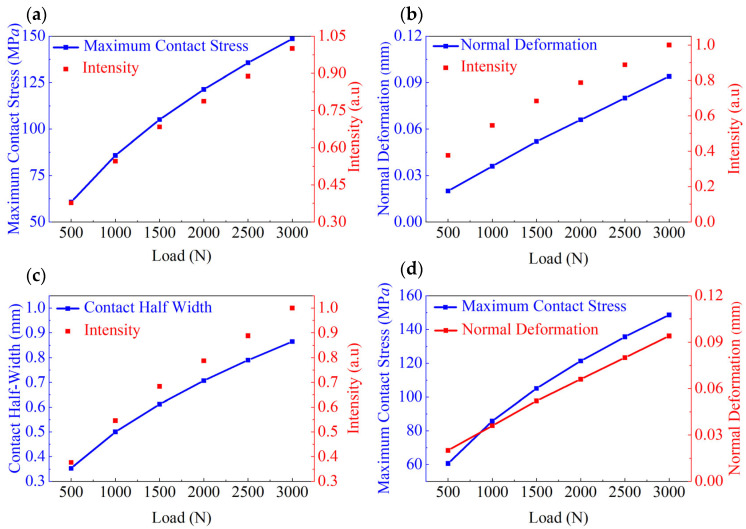
Parameters obtained at applied normal forces of 500–3000 N: (**a**) maximum contact stress and normalized light intensity, (**b**) normal deformation and normalized light intensity, (**c**) contact half-width and normalized light intensity, and (**d**) maximum contact stress and normal deformation.

Formula (5) indicates that the maximum contact stress is proportional to the square root of the normal force *F* and inversely proportional to the square root of the product of length *l* and radius *r*. Figure 1 illustrates the variations in several key parameters of the ML cylinder under different normal forces, including the maximum contact stress calculated based on Hertzian contact theory, and light intensity at the corresponding points measured experimentally. Figure 1a presents the values of the maximum contact stress (Equation (5)) and normalized light intensity for the applied normal forces ranging from 500 to 3000 N, showing a strong correlation between these two quantities and indicating that the light intensity can effectively measure the maximum contact stress. Figure 1b displays the normal deformation (Equation (6)) and light intensity during compression as functions of the load. Figure 1c depicts the contact half-width (Equation (1)) and light intensity under different normal forces. Figure 1d shows the contact stress and normal deformation under various normal forces. Figure S1 displays the wavelength and normalized light intensity obtained under different normal forces.

**Figure 2:**
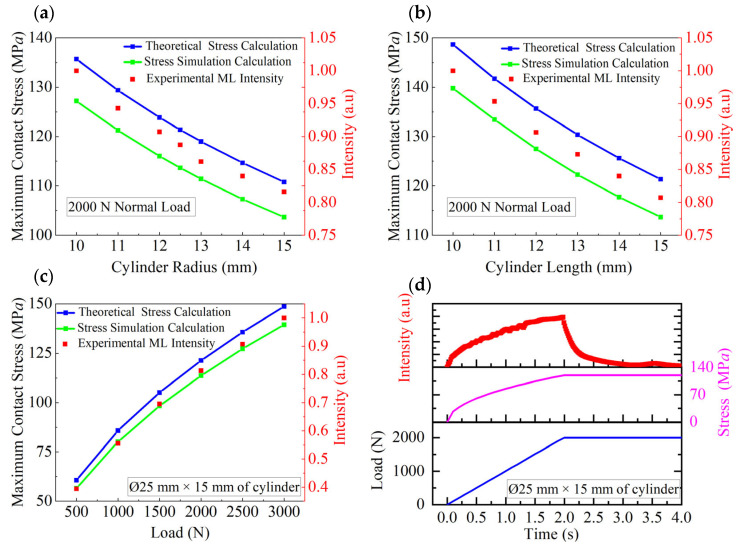
Maximum contact stress and corresponding light intensity plots constructed for ML cylinders. (**a**) Cylinders with various radii under a normal load of 2000 N. (**b**) Cylinders with various lengths under a normal load of 2000 N. (**c**) Cylinders under different normal loads (fixed: *l* = 15 mm, *r* = 12.5 mm). (**d**) Time-dependent curves obtained under incremental normal loading (fixed: *l* = 15 mm, *r* = 12.5 mm).

Figure 2a compares the maximum contact stress and light intensity at the corresponding location obtained for the ML cylinders with length *l* = 15 mm and various radii under a normal load of 2000 N. The theoretical results for the maximum contact stress, calculated using Equation (5), are represented by the blue line. The simulated results for the maximum contact stress, derived from finite element analysis are depicted by the green line. The experimental results shown by the red points represent the light intensity measured at the position of maximum contact stress. As cylinders radii increases, all results demonstrate similar variation trends. Figure 2b shows the relationship between the theoretical and simulated maximum contact stress and experimental light intensity obtained for the ML cylinders with a radius of 12.5 mm and various lengths under a normal load of 2000 N. As cylinder length increases, both the maximum contact stress and light intensity results demonstrate similar variation trends. Figure 2c compares the theoretical and simulated maximum contact stress and experimental light intensity for the ML cylinders with length *l* = 15 mm and radius *r* = 12.5 mm under different normal forces. These results demonstrate similar variation trends. Figure 2d displays the theoretical maximum contact stress and experimental light intensity variations under incremental loading (0–2000 N). As the force linearly increases, both the maximum contact stress and light intensity results demonstrate similar variation trends. Notably, the experimental light intensity data can be almost perfectly scaled to overlap with the theoretical and simulated maximum contact stress data. At 2000 N, the stress remains constant, whereas the light intensity at the maximum contact stress point decays rapidly over time. Figure S2 shows the light intensity variations observed for the ML cylinders of different sizes under loads of 500–3000 N. Figure S2a depicts the light intensities at the maximum contact stress point for the ML cylinders with length *l* = 15 mm and radii of 10–15 mm, while Figure S2b displays the intensity at the maximum contact stress point for the cylinders with a radius of 12.5 mm and lengths of 10–15 mm. These results indicate that under the same load, the light intensity at the maximum contact stress point decreases with increasing cylinder radius and length.

When the crosshead applies a normal load to the cylinder without a horizontal component or sliding, friction can be neglected. To simplify the calculations, this study ignored both friction and the tangential stress generated by cylinder deformation. At the area of contact, the stress state is primarily dominated by the normal stress, which reaches its peak value at the center of the contact area, known as the maximum contact stress. According to the von Mises stress formula [40,41], when only normal stress is present, the equivalent stress at the point of maximum contact stress is determined by this normal stress. Therefore, under these idealized conditions, the maximum contact stress can be approximated to be equal to the equivalent stress at that location. This equality holds specifically because the tangential components are negligible, and the stress state is largely controlled by the normal load. Thus, in a frictionless contact scenario, the equivalent stress at the maximum contact point can be approximated using the following formula.

(7) $$\sigma_{eq}=\sqrt{\frac{1}{2}[{\left(\sigma_{1}-\sigma_{2}\right)}^{2}+{\left(\sigma_{2}-\sigma_{3}\right)}^{2}+{\left(\sigma_{3}-\sigma_{1}\right)}^{2}]}$$

where *σ*_1_, *σ*_2_, and *σ*_3_ represent the three principal stresses in the contact region: *σ*_1_ is the normal stress at the center of the contact area, which is the maximum principal stress along the loading direction applied by the crosshead; *σ*_2_ is the stress in the radial direction along the contact surface; and *σ*_3_ is the stress in the tangential direction along the circumference of the contact area. Under frictionless conditions at the point of maximum contact stress, the tangential and transverse stresses are negligible, therefore $\sigma_{1}=\sigma_{max}$.

**Figure 3:**
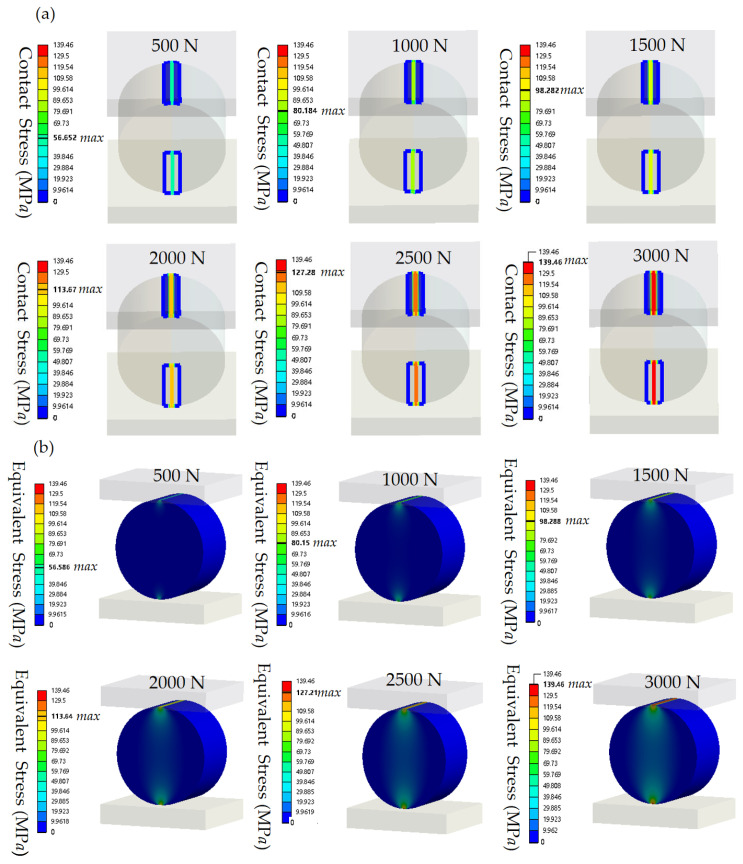
Finite element simulated stress contours of the ML cylinder (*r* = 12.5 mm, *l* = 15 mm) obtained under normal loads ranging from 500 to 3000 N. (**a**) Contact stress contours and (**b**) equivalent stress contours.

Figure 3 shows the stress contours from finite element simulations of an ML cylinder with a radius *r* = 12.5 mm and a length *l* = 15 mm obtained under normal compression. Figure 3a presents the contact-stress distribution contour for loads ranging from 500 to 3000 N, while Figure 3b displays the equivalent stress distribution under the same conditions. Supplementary Figures S3 and S4 further show the finite element simulated stress contours obtained for the ML cylinders under a load of 2000 N. In particular, Figure S3a,b display the contact stress and equivalent stress for the cylinders with radius *r* = 12.5 mm and lengths *l* = 10–15 mm, while Figure S4a,b depict the stress distributions obtained for the cylinders with length *l* = 15 mm and radii *r* = 10–15 mm. The simulation contours reveal the stress distributions under different load conditions and cylinder sizes, indicating that the maximum contact stress and the equivalent stress are nearly identical under uniaxial normal loading.

**Figure 4:**
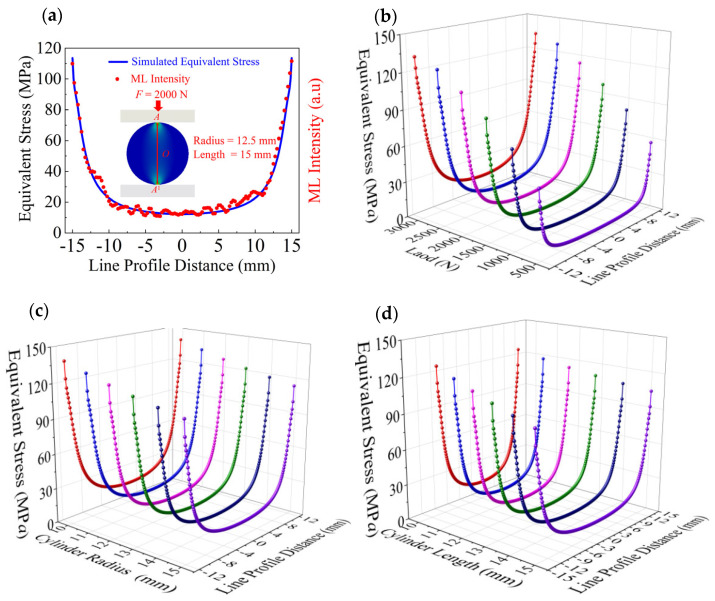
Stress–partial light intensity distributions along path *A*–*A*^1^ of the ML cylinders. (**a**) Relationship between the simulated equivalent stress and experimentally measured light intensity along path *A*–*A*^1^ obtained for the ML cylinder with *r* = 12.5 mm and *l* = 15 mm under a normal load of 2000 N. (**b**) Simulated equivalent stress contours obtained for the ML cylinder with *r* = 12.5 mm and *l* = 15 mm under normal loads ranging from 500 to 3000 N. (**c**) Simulated equivalent stress distributions along path *A*–*A*^1^ obtained for the ML cylinders with *l* = 15 mm and *r* = 10–15 mm under a normal load of 2000 N; (**d**) Simulated equivalent stress distributions along path *A*–*A*^1^ obtained for the ML cylinders with *r* = 12.5 mm and *l* = 10–15 mm under a normal load of 2000 N.

To investigate the stress distribution within the ML cylinder as the contact stress propagates into its interior, the *A*–*A*^1^ path segment was selected for focused analysis. Figure 4 shows the equivalent stress and partial light intensity distribution along the *A*–*A*^1^ path for the ML cylinders. Figure 4a presents the simulated equivalent stress distribution along the *A*–*A*^1^ path for the ML cylinder with *r* = 12.5 mm and *l* = 15 mm obtained under a normal load of 2000 N, and the experimentally measured light intensity distribution is also shown, revealing a close correlation between the equivalent stress and the light intensity. Figure 4b shows the *A*–*A*^1^ simulated equivalent stress distributions along the *A*–*A*^1^ path under normal loads ranging from 500 to 3000 N for the same cylinder size under different loads, which shows that the stress increases with the applied force. Figure 4c displays the simulated equivalent stress distribution along the *A*–*A*^1^ for the ML cylinders with *l* = 15 mm and *r* = 10–15 mm under a normal load of 2000 N, which shows that the stress decreases with the increasing radius of the cylinder. Figure 4d shows the simulated equivalent stress distributions along the *A*–*A*^1^ path for the ML cylinders with *r* = 12.5 mm and *l* = 10–15 mm under a normal load of 2000 N, which indicates that the equivalent stress decreases with the increasing length of the cylinder.

Figure 4a shows the simulated equivalent stress and the experimentally measured normalized light intensity distribution along section *A*–*A*^1^. A close similarity is observed between the normalized light intensity and the equivalent stress distribution, aligning with findings from similar experiments in the literature [23]. Within the framework of CaZnOS-based systems research, recent studies [42] have investigated the stress and light intensity distributions of ML cylinders with and without defects under various normal loads, revealing a significant similarity between the two. This finding provides critical evidence for understanding the intrinsic relationship between ML intensity and stress. Additionally, this relationship has been further validated through our analytical and experimental investigations, as expressed by the following formula:

(8) $$\sigma_{eq}=\frac{1}{k}\cdot I$$

where *σ_eq_* is the equivalent stress, and 1/*k* is the proportionality factor. Because relative intensities rather than absolute light intensities are typically reported in the literature [1], it is not reasonable to compare the actual light intensity. Thus, the proportionality factor 1/*k* depends on the measurement equipment and testing method.

Moreover, we note that this relationship holds under the condition that the measured luminescence intensity exceeds the material’s minimum sensitivity threshold yet remains below saturation.

### 3.2. Effect of Stress Rate

In ML cylinder compression experiments, the stress rate $\dot{\sigma }$ is directly related to the rate of change in the applied normal force $\dot{F}$. The relationship between the rate of change in the maximum contact stress $\sigma_{max}$ and rate of change in the normal force *F* is expressed by the following formula:

(9) $$\;\dot{\sigma_{max}}=\frac{d\sigma_{max}}{dt}\;=\frac{d}{dt}(\sqrt{\frac{F*E^{*}}{\pi *r*l}})\;=\frac{1}{2}(\sqrt{\frac{E^{*}}{\pi *r*l}})\cdot \frac{1}{\sqrt{F}}\cdot \frac{dF}{dt}$$

This relationship shows how the maximum contact stress rate $\dot{\sigma_{max}}$ relies upon the normal force rate $\dot{F}$. By adjusting $\dot{F}$, the maximum contact stress rate of the ML cylinder can be controlled, influencing its luminescence intensity. Because the light intensity *I* is proportional to the equivalent stress *σ_eq_*, and the maximum contact stress is equal to the equivalent stress at that location, the following relationship between the light intensity rate $\dot{I}$ and the maximum contact stress rate $\dot{\sigma }$ can be derived.

(10) $$\dot{I}=\frac{dI}{dt}=\frac{d(K\dot{\sigma_{max}})}{dt}=k\;\cdot \dot{\sigma_{max}}$$

By combining Equations (9) and (10), the following expression is obtained:

(11) $$\frac{dI}{dt}=\frac{1}{2}k\cdot (\sqrt{\frac{E^{*}}{\pi *r*l}})\cdot \frac{1}{\sqrt{F}}\cdot \frac{dF}{dt}\;=M\cdot \frac{1}{\sqrt{F}}\cdot \frac{dF}{dt}$$

Or:

(12) $$I=M\int \frac{1}{\sqrt{F}}dF\;I=I_{0}+2M\cdot \sqrt{F}$$

where *I*_0_ represents the initial light intensity. M is a constant related to the cylinder size, effective modulus, and light-measurement setup. Thus, under the same normal compression, the light intensity rate $\dot{I}$ is proportional to the load rate $\dot{F}$. A faster load rate results in a more significant change in the light intensity. However, at different normal loads, the same loading rate leads to a slower change in the light-intensity rate with increasing load force.

By combining Equations (1) and (6), it can be seen that the normal force *F* and normal deformation δ are approximately linearly related, while the crosshead speed is proportional to the normal deformation. Therefore, we can assume that the rate of change in applied force (loading rate) *dF*/*dt* is proportional to the crosshead speed *v*:

(13) $$\frac{dF}{dt}=n\cdot v$$

where *n* is a constant related to the material and geometric properties. By combining Equations (11) and (13), we can further analyze the relationship between the light intensity *I* and the crosshead speed *v*:

(14) $$\frac{dI}{dt}=(M\cdot n)\cdot \frac{v}{\sqrt{F}}$$

To determine the relationship between light intensity *I* and crosshead speed *v*, we integrate the light intensity rate with respect to time *t*; assuming that the applied force *F* eventually stabilizes, *F* can be approximated as constant, allowing 1/$\sqrt{F}$ to also be treated as approximately constant. Under these conditions, the light intensity rate is proportional to the crosshead speed:

(15) I∝v·t

This indicates that, as the applied force stabilizes, the light intensity *I* is proportional to the crosshead speed *v*; thus, a higher crosshead speed results in faster light intensity accumulation and a greater final light intensity.

**Figure 5:**
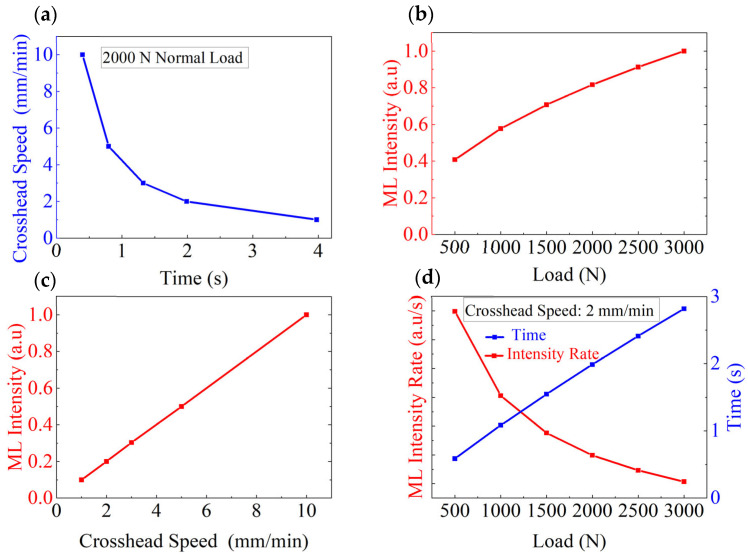
Relationships between the light intensity, the light intensity rate, and various parameters under different loading paths. (**a**) Crosshead Speed vs. time up to 2000 N; (**b**) applied force vs. normalized light intensity; (**c**) crosshead speed vs. normalized light intensity per unit of time. (**d**) Applied force vs. normalized light intensity rate at a crosshead speed of 2 mm/min.

Figure 5 illustrates the dependencies of light intensity, light intensity rate, and various parameters under different loading paths. An ML cylinder with a radius of 12.5 mm and length of 15 mm was selected as the research object. Figure 5a displays the relationship between the crosshead speed and loading duration during normal loading up to 2000 N. This relationship is derived by combining the normal deformation equation of the cylinder after contact with the crosshead, as given by Equation (6), with the loading rate. The crosshead speed represents the rate of movement of the testing machine’s crosshead and determines the time needed to reach the specified load. Table S1 lists the specific times required for different crosshead speeds under varying normal loading forces, also derived from Equation (6). Figure 5b shows the relationship between the normalized light intensity and the applied force based on Equation (12). As the applied force increases, the rate of increase in light intensity declines. Figure 5c displays a linear relationship between the crosshead speed and normalized light intensity per unit of time based on Equation (14), which indicates that the crosshead speed is positively correlated with the light intensity. Figure 5d depicts the relationship between the normal load and light intensity rate obtained at a crosshead speed of 2 mm/min based on Equation (11). The blue curve represents the dependence of load on loading time, derived from Table S1, while the red line segment indicates the light intensity rate. As the load increases, the light intensity rate decreases.

**Figure 6:**
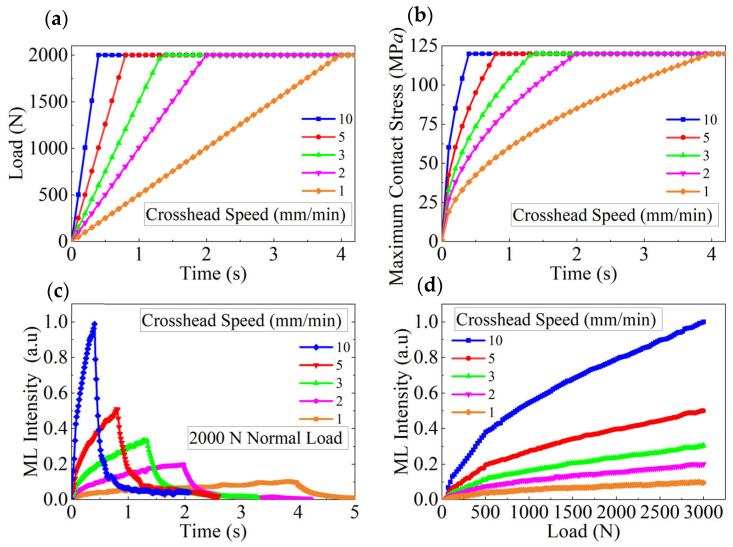
Relationships at different crosshead speeds. (**a**) Load versus loading time plotted at different crosshead speeds. (**b**) Maximum contact stress versus loading time at 2000 N plotted at different crosshead speeds. (**c**) Loading time versus normalized light intensity at 2000 N plotted at different crosshead speeds. (**d**) Applied force versus normalized light intensity plotted at different crosshead speeds.

Figure 6 shows the relationships at different crosshead speeds. Figure 6a depicts the plot of the applied force versus the loading time calculated at different crosshead speeds (1–10 mm/min). Note that in this study, the maximum normal force was set to 2000 N, and once it was reached, the crosshead stopped descending, maintaining the load. Figure 6b shows the plots of the maximum contact stress versus time, calculated under the same conditions using Equation (5). The results indicate that regardless of the crosshead speed, the maximum contact stress is the same at 2000 N. Figure 6c shows the plots of the experimental normalized light intensity versus time with higher crosshead speed resulting in faster and greater intensity changes. This phenomenon can be explained as follows: as the loading rate increases, the internal piezoelectric field of the material strengthens [8]. These traps capture and temporarily store charge carriers (electrons and holes). During stress stimulation, the carriers are rapidly released, recombine, and emit photons. At higher stress rates, the excitation and release of carriers are accelerated, increasing the number of electron transitions and luminescence intensity. Thus, a higher loading rate results in a higher stress rate and a more pronounced luminescence response. In addition, the plots in Figure 6b,c are closely similar slopes during the stress increase phase, indicating that the luminescence intensity is correlated with the pressure per unit area. Once the applied force reaches its maximum and stabilizes, the light intensity rapidly decreases. Figure 6d shows the plots of the applied normal force versus the experimental normalized light intensity constructed at different crosshead speeds, indicating that the intensity depends on both the loading rate and the magnitude of the applied force. When the crosshead speed is constant, the rate of increase in light intensity declines as the applied force increases.

### 3.3. Relationship Between Light Intensity and Strain Energy Density of the ML Cylinder

During compression, the strain energy is stored in the deformed material, which is absorbed during deformation and released when the stress is removed. For elastic deformation, the strain energy is fully reversible. For linear elastic materials, the total strain energy *U* is calculated by integrating the product stress and strain over the entire volume of the material [43]. Owing to the complex distributions of both stress and strain in the ML cylinder, we focus exclusively on the relationship between the strain energy density at the maximum contact stress point and the corresponding light intensity at that point. The maximum contact stress *σ_max_* is equal to the equivalent stress *σ_eq_* at that location according to Equation (7) and according to Equation (8) the equivalent stress *σ_eq_* is proportional to the light intensity *I*. The strain energy density is determined by *σ_max_*, normal force *F*, and geometric parameters of the ML cylinders, and the strain energy density at the maximum contact stress point *U_max_* is expressed as:

(16) $$U_{max}=\frac{1}{2}\sigma_{max}\cdot \varepsilon_{max}$$

where $\varepsilon_{max}$ is the maximum contact strain. For linear elastic materials, the stress and strain are correlated via Young’s modulus $E_{1}$:

(17) $$\sigma_{max}=E_{1}\cdot \varepsilon_{max}$$

Substituting this relationship into the strain energy density formula yields

(18) $$U_{max}=\frac{\sigma_{max}^{2}}{2E_{1}}$$

Substituting Equation (5) for *σ_max_*, we have

$$U_{max}=\frac{F\cdot E^{*}}{2E_{1}\cdot \pi \cdot r\cdot l}$$

Combining Equations (7), (8), and (18), we obtain

(19) $$I_{max}=k\cdot \sqrt{2E_{1}\cdot \;U_{max}}$$

**Figure 7:**
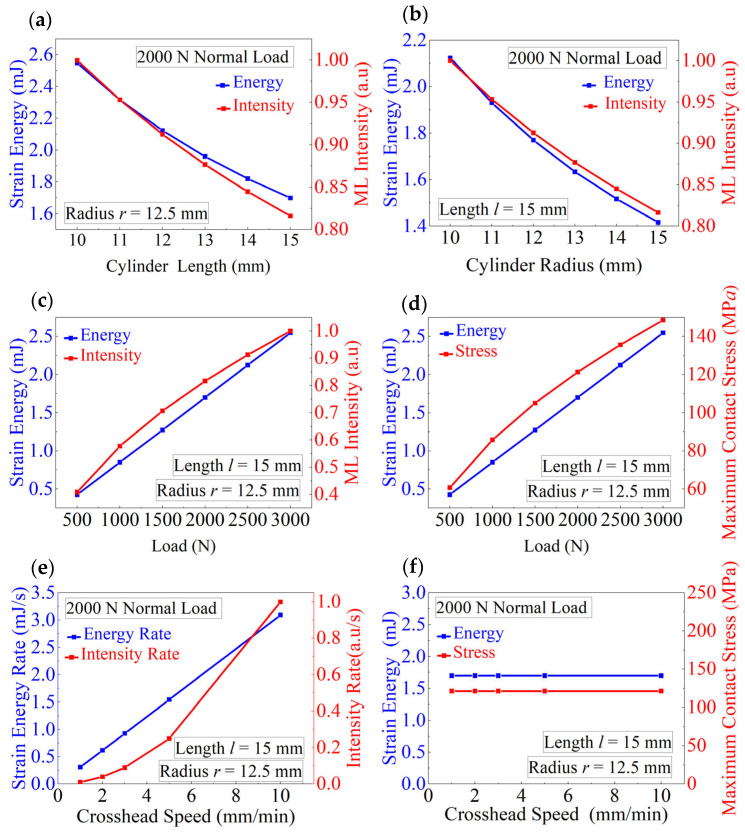
Relationships between the strain energy, strain energy rate, and other parameters determined for the ML cylinders of different sizes under various loading paths. (**a**) Light intensity as a function of strain energy plotted for the ML cylinders of different lengths. (**b**) Light intensity as a function of strain energy plotted for the ML cylinders of different radii. (**c**) Light intensity as a function of strain energy plotted at different applied forces. (**d**) Maximum contact stress as a function of strain energy plotted at different forces. (**e**) Light intensity rate as a function of strain energy rate plotted at different crosshead speeds. (**f**) Maximum contact stress as a function of strain energy plotted at different crosshead speeds.

Equation (19) implies that the light intensity *I_max_* at the point of maximum contact stress is proportional to the square root of strain energy density *U*. As the strain energy at the point of maximum contact stress increases, the light intensity at that point increases proportionally to the square root of the strain energy. This relationship reveals a key feature of ML materials: the luminescence depends not only on the loading path and contact conditions, but also on the cylinder size, and energy storage capacity. To verify this conclusion, compression tests were conducted on ML cylinders under a normal load of 2000 N, focusing on analyzing the maximum contact stress point at the center of the contact region. Figure 7a shows that for a cylinder with a radius of 12.5 mm, both the strain energy (calculated using Equation (17)) and light intensity (experimentally measured) decrease as the cylinder length increases because the maximum contact stress (calculated using Equation (5)) decreases with increasing length. Figure 7b shows a similar trend observed for a cylinder with *l* = 15 mm and different radii: as the radius increases, both the light intensity and strain energy decrease. Figure 7c,d further illustrate the variations of the strain energy, ML intensity, and maximum contact stress under different loads.

For ML materials, the rate of change of strain energy is a key parameter for characterizing internal energy variations over time, which significantly influences the light intensity according to experimental observations. The rate of change of strain energy density at the maximum contact stress is expressed as

(20) $$\dot{u}=\frac{d}{dt}(\frac{\sigma_{max}^{2}}{2E_{1}})\;=\frac{\sigma_{max}}{E_{1}}\cdot \frac{d\sigma_{max}}{dt}\;\;\;=\frac{I_{max}}{k^{2}\cdot E_{1}}\cdot \frac{dI_{max}}{dt}\;=Q\cdot I_{max}\cdot \frac{dI_{max}}{dt}$$

where Q is a proportional constant that is related to the elastic modulus of the ML cylinder and the method used for detecting light intensity.

Equation (20) shows that the strain energy change rate is directly proportional to the product of the light intensity and rate of change. Thus, by measuring the light intensity and its rate of change at the contact center region, the strain energy density rate at that region can be determined. Figure 7e,f display the variations of the change rates of strain energy and the light intensity, the strain energy, and the maximum contact stress as function of the crosshead speed. A linear relationship is observed between the change rate of strain energy and the crosshead speed, while the change rate of light intensity increases with increasing crosshead speed non-linearly. However, both the maximum contact stress and strain energy remain constant.

As the rate of change of strain energy increases, the strain energy within the epoxy resin matrix rapidly accumulates and is transferred to the embedded ML material, leading to greater energy activation of electrons and resulting in stronger light intensity.

When an ML material is in a static state, the deformation of the cylinder remains unchanged, and the epoxy resin matrix stops transferring energy to the stress sensor (ML material). If no new external forces are applied or removed, both stress σ and strain ε remain constant, thus the strain energy in the material does not change. According to Equation (19), since the strain energy rate is proportional to the product of the light intensity rate and light intensity, either the light intensity or its rate of change must be zero. However, when the external force stops changing, the light intensity does not instantly drop to zero. As a result, the rate of change of light intensity gradually decreases until it eventually becomes zero. Thus, without further mechanical energy input, the light intensity initially remains stable. Nevertheless, due to the afterglow effect, the light intensity of the ML material gradually decays over time, which is independent of the external force application. This is a natural process of internal energy release within the material. The energy stored in the internal trap states is slowly released as photons, causing the material to glow for a certain time. The electrons in the trap states are gradually released or depleted through non-radiative recombination, decreasing the light intensity until it becomes imperceptible. The afterglow can be described using the following formula [44,45]:

(21) $$I(t)=(I_{max}-A_{1}-A_{2})+A_{1}\;exp(-t/\tau_{1})+A_{2}\;exp(-t/\tau_{2})\;$$

where *I*(*t*) is the light intensity at time *t*, and *I_max_* is the maximum intensity. *A*_1_ and *A*_2_ are the fitting coefficients representing the contributions of the two decay processes. *τ*_1_ and *τ*_2_ are the corresponding decay time constants reflecting different decay rates.

**Figure 8:**
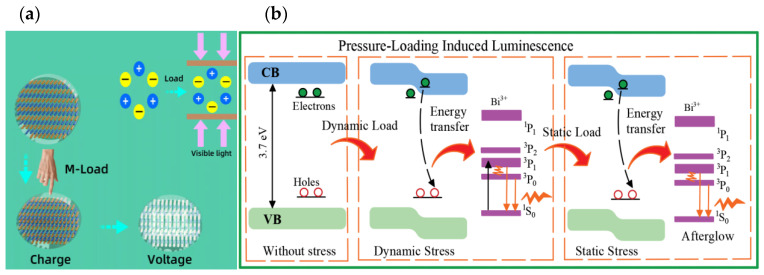
Schematics of the luminescence process in CaZnOS. (**a**) Internal electric field generated in CaZnOS and (**b**) ML mechanism of Bi^3+^ ions in CaZnOS.

When the crosshead compresses an ML cylinder, the phenomena depicted in Figure 8 are observed. Figure 8a shows that without an external force, the charge centers of the cation and anion coincide. Upon application of a force, the charge centers shift slightly, generating a dipole moment [25]. The dipole moments of all crystal units in the stressed region combine to form a macroscopic internal electric field along the stress direction, inducing a local bandgap change. Figure 8b shows that electrons near the conduction band are released under stress, enter the conduction band, and then recombine non-radiatively with holes. The released energy is transferred to Bi^3+^ ions, generating blue–green emission through the ^3^P_1,0_ → ^1^S_0_ transition. This phenomenon demonstrates that the conversion of mechanical energy into light is driven by the dynamic stress, which excites and releases electrons and holes.

Once the normal pressure is stabilized, the deformation of the cylinder reaches a static state, and the stress and strain of the material stop changing, halting the further energy conversion into light and ceasing the electron excitation. Although some excited-state electrons will transit and emit light, it fades as the afterglow effect diminishes and ultimately ceases. This indicates that under static stress, ML materials cannot sustain emission, and the light intensity becomes zero. When the compressive force is released, the elastic recovery of the ML cylinder releases the stored strain energy, and the resulting dynamic stress triggers another burst of light similar to the second energy release, causing light emission by the ML cylinder.

## 4. Conclusions

We conducted a comprehensive study on mechanoluminescent (ML) cylinders under normal loading, investigating the interplay among stress distribution, luminescence behavior, and strain energy. By integrating theoretical derivations, finite element simulations, and experimental validations, we established a near-proportional relationship between the maximum contact stress and luminescence intensity during elastic deformation. This study demonstrated that the equivalent stress at the contact center is a reliable indicator of light emission. A more accurate representation of ML behavior under complex stress conditions is provided. Notably, the dimensions of ML cylinders, including length and radius, significantly influence internal stress distribution and luminescence signals. Compared to the findings in previous literature, this study further quantifies the impact of geometric parameters on stress and light intensity. This approach provides a viable method for indirectly estimating an object’s geometry through optical measurements. Furthermore, our investigation clarified the role of dynamic strain energy accumulation in governing the real-time luminescent response, particularly at various loading rates. We observed that higher rates of strain energy change result in higher luminescence intensity, whereas static or equilibrium conditions lead to nonluminescent or fading signals. Additionally, we introduced a novel model linking strain energy density and its rate of change to luminescence intensity. This model offers a theoretical framework to explain why ML materials remain nonluminescent under static conditions yet emit light during dynamic loading. These insights elucidate the dynamic mechanisms driving luminescence, offering an alternative explanation for the static non-luminescent or luminescence decay phenomena reported in previous literature.

In this study, by integrating strain energy dynamics and geometric effects, a more comprehensive explanation for ML behavior across various loading paths with different object sizes and stress scenarios is established.

In future work, efforts could focus on investigating the relationship between luminescence thresholds, maximum saturation intensity, applied load, and stress in mechanoluminescent (ML) materials under dynamic loading conditions, aiming to optimize performance and expand potential applications.

Innovations and Highlights

1. **Stress and light intensity correlation:** This study systematically investigates for the first time the maximum contact stress and light intensity at the corresponding location for ML cylinders under normal loading, discovering that these distributions show good similarity under different normal forces. In combination with theoretical and simulated stress analysis, along with light-intensity experiments under different sample sizes and loading paths, it establishes the stress–light intensity relationship to deepen our understanding of ML material properties under complex conditions.
2. **Dynamic and static conditions:** The luminescent responses of the Ca(1–2*x*%)ZnOS%Bi^3+^, *x*%Li^+^ (*x* = 5) ML material under both the dynamic and static stresses are systematically studied, which establishes a foundation for their applications in various stress environments.
3. **Geometry and luminescent response:** This study systematically analyzes for the first time the impact of geometric dimensions (such as length and radius) on the stress distribution and luminescence intensity of ML cylinders, revealing the significant role of geometric parameters in the optical response of ML materials. Based on the analysis of ML luminescence response, it is theoretically possible to infer the geometric dimensions of the measured object, enabling indirect measurement of its shape and structural features.
4. **Strain Energy and Luminescent Response:** This study establishes, for the first time, a model linking strain energy density and its rate of change to light intensity and its variation rate, clarifying why ML materials remain non-luminescent under static loads yet emit light under dynamic loads. Additionally, it reveals that elastic recovery following unloading can induce secondary luminescence, offering theoretical support for the application of ML materials in dynamic stress monitoring.
5. **Broad application range:** This study provides a theoretical support for the development of high-precision and high-sensitivity sensors based upon ML materials and used, for instance, in structural health monitoring, flexible electronics, and smart sensors.

## Data Availability

The original contributions presented in the study are included in the article/Supplementary Materials. Further inquiries can be directed to the corresponding authors.

## Supplementary Materials

The following supporting information can be downloaded at: https://www.mdpi.com/article/10.3390/ma18020331/s1. Figure S1. Wavelengths and normalized light intensities obtained under different normal forces. Figure S2. Light intensities at the maximum contact stress location obtained for the ML cylinders of different sizes under normal forces of 500–3000 N. Figure S3. Finite element simulated stress contours of the ML cylinders of different sizes under a normal load of 2000 N. Figure S4. Finite element simulated stress contours of the ML cylinders of different sizes under a normal load of 2000 N. Table S1. Loading times required for the ML cylinders with different loading paths.

## References

[1] Zhang J.C., Wang X., Marriott G., Xu C.-N. (2019). Trap-controlled mechanoluminescent materials. Prog. Mater. Sci..

[2] Li W., Cai Y.Y., Chang J.Q., Wang S.S., Liu J.J., Zhou L., Wu M.M., Zhang J.-C. (2023). Unraveling mechanoluminescent mechanisms in doped CaZnOS materials: Co-mediation of trap-controlled and non-trap-controlled Processes. Adv. Funct. Mater..

[3] Zhuang Y., Xie R.-J. (2024). Mechanoluminescence rebrightening the prospects of stress sensing: A review. Adv. Mater..

[4] Xiao Y., Xiong P., Zhang S., Sun Y., Yan N., Wang Z., Chen Q., Shao P., Brik M.G., Ye S. (2023). Cation-defect-induced self-reduction towards efficient mechanoluminescence in Mn^2+^-activated perovskites. Mater. Horiz..

[5] Wang W., Tang K.W.K., Pyatnitskiy I., Liu X., Shi X., Huo D., Jeong J., Wynn T., Sangani A., Baker A. (2023). Ultrasound-induced cascade amplification in a mechanoluminescent nanotransducer for enhanced sono-optogenetic deep brain stimulation. ACS Nano.

[6] Mohamed A.Y.A., Welles L., Siggins A., Healy M.G., Brdjanovic D., Rada-Ariza A.M., Lopez-Vazquez C.M. (2020). Effects of substrate stress and light intensity on enhanced biological phosphorus removal in a photo-activated sludge system—Science direct. Water Res..

[7] Ishida S., Narahara A. (2019). Stress-rate effect on time response of mechanoluminescent-sensor luminescent intensity. Opt. Express.

[8] Yang H., Wei Y., Ju H., Huang X., Li J., Wang W., Peng D., Tu D., Li G. (2024). Microstrain-stimulated elastico-mechanoluminescence with dual-mode stress sensing. Adv. Mater..

[9] Chen B., Zhang X., Wang F. (2021). Expanding the toolbox of inorganic mechanoluminescence materials. Acc. Mater. Res..

[10] Wang W., Wang Z.-B., Zhang J., Zhou J., Dong W., Wang Y. (2022). Contact electrification induced mechanoluminescence. Nano Energy.

[11] Xin Q., Cai Z., Su M., Li F., Fang W. (2018). Printable skin-driven mechanoluminescence devices via nanodoped matrix modification. Adv. Mater..

[12] Xie Z., Xue Y., Zhang X., Chen J., Lin Z., Liu B. (2024). Isostructural doping for organic persistent mechanoluminescence. Nat. Commun..

[13] Yue Y., Pan G., Wan J., Xiao Z., Zhang Y., Xu S., Bai G. (2024). Smart films based on semiconductor heterojunctions of mechanoluminescent sulfide and sulfur oxide for stress sensing and anti-counterfeiting applications. Chem. Eng. J..

[14] Du Y., Jiang Y., Sun T., Zhao J., Huang B., Peng D., Wang F. (2019). Mechanically excited multicolor luminescence in lanthanide ions. Adv. Mater..

[15] Cai Y., Liu S., Zhao L., Wang C., Lv H., Liu B., Qiu J., Xu X., Yu X. (2022). Delayed stress memory by CaAl_2_O_4_:Eu^2+^ mechanoluminescent phosphor with defect engineering regulation. J. Adv. Ceram..

[16] Petit R.R., Michels S.E., Feng A., Smet P.F. (2019). Adding memory to pressure-sensitive phosphors. Light Sci. Appl..

[17] Xiao Y., Wei X., Wang S., Yang K., Li S. (2016). Dynamic triboelectrification-Induced electroluminescence and its use in visualized sensing. Adv. Mater..

[18] Li M., Chen H., Zhao J., Xia M., Xing Q., Wang W., Liu Q., Lu Y., Luo M., Zhu X. (2024). Stretchable continuous p-n alternating thermoelectric fibers for energy harvesting and sensing devices. Adv. Compos. Hybrid Mater..

[19] Chen C., Zhuang Y., Li X., Lin F., Peng D., Tu D., Xie A., Xie R.-J. (2021). Achieving remote stress and temperature dual-modal imaging by double-lanthanide-activated mechanoluminescent materials. Adv. Funct. Mater..

[20] Chen C., Lin Z., Huang H., Pan X., Zhou T.-L., Luo H., Jin L., Peng D., Xu J., Zhuang Y. (2023). Revealing the intrinsic decay of mechanoluminescence for achieving ultra-fast-response stress sensing. Adv. Funct. Mater..

[21] Li Z.-B. (2020). Force-induced 1540 nm luminescence: Role of piezotronic effect in energy transfer process for mechanoluminescence. Nano Energy.

[22] Fujio Y., Xu C.-N., Terasawa Y., Sakata Y., Yamabe J., Ueno N., Terasaki N., Yoshida A., Watanabe S., Murakami Y. (2016). Sheet sensor using SrAl_2_O_4_:Eu mechanoluminescent material for visualizing inner crack of high-pressure hydrogen vessel. Int. J. Hydrogen Energy.

[23] Yang X., Liu R., Xu X., Liu Z., Sun M., Yan W., Peng D., Xu C.-N., Huang B., Tu D. (2021). Effective repeatable mechanoluminescence in heterostructured Li_1−x_Na_x_NbO_3_: Pr^3+^. Small.

[24] Yang Y.-L., Li T., Guo F., Yuan J.-Y., Zhang C.-H., Zhou Y., Li Q.-L., Wan D.-Y., Zhao J.-T., Zhang Z.-J. (2022). Multiple color emission of mechanoluminescence and photoluminescence from SrZnSO:Bi^3+^ for multimode anticounterfeiting. Inorg. Chem..

[25] Yang Y.-L., Yang X.-C., Yuan J.-Y., Li T., Fan Y.-T., Wang L., Deng Z., Li Q.-L., Wan D.-Y., Zhao J.-T. (2021). Time-resolved bright red to cyan color tunable mechanoluminescence from CaZnOS: Bi^3+^, Mn^2+^ for anti-counterfeiting device and stress sensor. Adv. Opt. Mater..

[26] Yuan J.-Y., Yang Y.-L., Yang X.-C., Fan Y.-T., Li T., Huang M., Zhang F., Li Q.-L., Zhao J.-T., Zhang Z.-J. (2021). Regulating the trap distribution to achieve high-contrast mechanoluminescence with an extended saturation threshold through co-doping Nd^3+^ into CaZnOS:Bi^3+^, Li^+^. J. Mater. Chem. C.

[27] Fan Y.-T., Yang Y.-L., Li T., Yuan J.-Y., Li Q.-L., Zhao J.-T., Wan D.-Y., Zhang Z.-J. (2021). Enhanced mechanically induced red-light emitting novel mechanoluminescence materials for ultrasonic visualization and monitoring applications. J. Mater. Chem. C.

[28] Zhou Y., Yang Y.-L., Fan Y.-T., Yang W., Zhang W.-B., Hu J.-F., Zhang Z.-J., Zhao J.-T. (2019). Intense red photoluminescence and mechanoluminescence from Mn^2+^-activated SrZnSO with a layered structure. J. Mater. Chem. C.

[29] Tu D., Xu C.-N., Yoshida A., Fujihala M., Hirotsu J., Zheng X.-G. (2017). LiNbO_3_:Pr^3+^: A multipiezo material with simultaneous piezoelectricity and sensitive piezoluminescence. Adv. Mater..

[30] Zhang J.-C., Xu C.-N., Kamimura S., Terasawa Y., Yamada H., Wang X. (2013). An intense elastico-mechanoluminescence material CaZnOS:Mn^2+^ for sensing and imaging multiple mechanical stresses. Opt. Express.

[31] Yang Y.-L., Li Q.-L., Yang X.-C., Yang W., An R., Li T., Zhou Y., Zhang H.-W., Zhao J.-T., Zhang Z.-J. (2020). Color manipulation from Bi^3+^-activated CaZnOS under stress with ultra-high efficiency and low threshold for multiple anticounterfeiting. J. Mater. Chem. C.

[32] Liu L., Xu C.-N., Yoshida A., Tu D., Ueno N., Kainuma S. (2019). Scalable elasticoluminescent strain sensor for precise dynamic stress imaging and onsite infrastructure diagnosis. Adv. Mater. Technol..

[33] Wen B.-C. (2018). Mechanical Design Manual.

[34] Gourgiotis P.-A., Zisis T., Giannakopoulos A.-E., Georgiadis H.-G. (2019). The hertz contact problem in couple-stress elasticity. Int. J. Solids Struct..

[35] Hertz H. (1881). Über die Berührung fester elastischer Körper. J. Reine Angew. Math..

[36] Liaudet J.P., Rigaud E. (2006). Response of an impacting hertzian contact to an order-2 subharmonic excitation: Theory and experiments. J. Sound Vib..

[37] Sayles R.S., Desilva G.M.S., Leather J.A., Anderson J.C., Macpherson P.B. (1981). Elastic conformity in hertzian contacts. Tribol. Int..

[38] Yang X.-Q. (2018). Contact Mechanics Theory and Rolling Bearing Design Analysis.

[39] Johnson K.L., Greenwood J.A. (1997). An adhesion map for the contact of elastic spheres. J. Colloid Interface Sci..

[40] Oluwole O., Emagbetere E. (2013). Finite element analysis of in-plane displacements and von-mises stresses in ell-ipsoidal and circular cylinderical petroleum tankers. Sci. Res..

[41] Matsui S. (2023). Practical estimation method for extreme value distribution of von Mises stress in ship structure. J. Mar. Sci. Technol..

[42] Sun C.Y., Liu W., Shi X., Rao G.H., Zhao J.T. (2025). Consistency of simulated stress distribution with experimental mechanoluminescent intensity distribution facilitating stress visualization device design. J. Lumin..

[43] Feng Y.-T., Gao W. (2021). On the strain energy distribution of two elastic solids under smooth contact. Powder Technol..

[44] Wei Y., Xing G.C., Liu K., Li G.G., Dang P.P., Liang S.S., Liu M., Cheng Z.Y., Jin D.Y., Lin J. (2019). New strategy for designing orangish-red-emitting phosphor via oxygen-vacancy-induced electronic localization. Light Sci. Appl..

[45] Chen Y., Zhang Y., Karnaushenko D., Chen L., Hao J., Ding F., Schmidt O.G. (2017). Addressable and Color-Tunable Piezophotonic Light-Emitting Stripes. Adv. Mater..

