# Supplementary material for: Stress Distributions and Luminescent Responses of Mechanoluminescent Cylinders with Various Sizes and Loading Paths
Source: Materials (Basel). 2025 Jan 13;18(2):331. doi: 10.3390/ma18020331 (PMC11766612; doi:10.3390/ma18020331)
Supplement: Supplementary file 1 [file materials-18-00331-s001.zip › materials-3382508-supplementary.pdf]

materials

Supporting Information

Stress Distributions and Luminescent Responses of Mechanoluminescent Cylinders with Various Sizes and Loading Paths

Chang-Ying Sun [1], Wei Liu [1], Xin Shi [2], Guang-Hui Rao [1,2,*] and Jing-Tai Zhao [1,2,*]

[1] School of Mechanical and Electrical Engineering, Guilin University of Electronic Technology, Guilin 541004, China; zyscy352016@163.com (C.-Y.S.); liuwei@mails.guet.edu.cn (W.L.)
[2] Guangxi Key Laboratory of Information Materials & School of Materials Science and Engineering, Guilin University of Electronic Technology, Guilin, 541004, China; shixin0324@163.com
* Correspondence: rgh@guet.edu.cn (G.-H.R.); jtzhao@guet.edu.cn (J.-T.Z.)

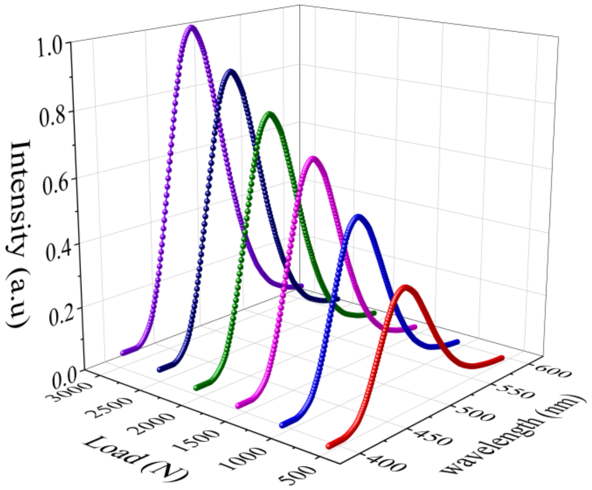

Figure S1. Wavelengths and normalized light intensities obtained under different normal forces.

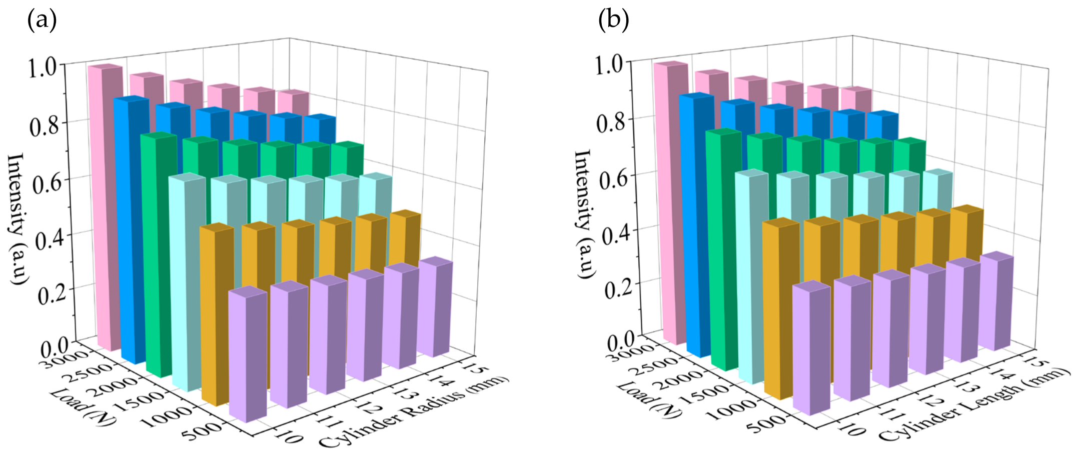

Figure S2. Light intensities at the maximum contact stress location obtained for the ML cylinders of different sizes under normal forces of 500–3000 N. a) Light intensities of the ML cylinders with a length of 15 mm and radius ranging from 10 to 15 mm. b) Light intensities of the ML cylinders with a radius of 12.5 mm and lengths ranging from 10 to 15 mm.

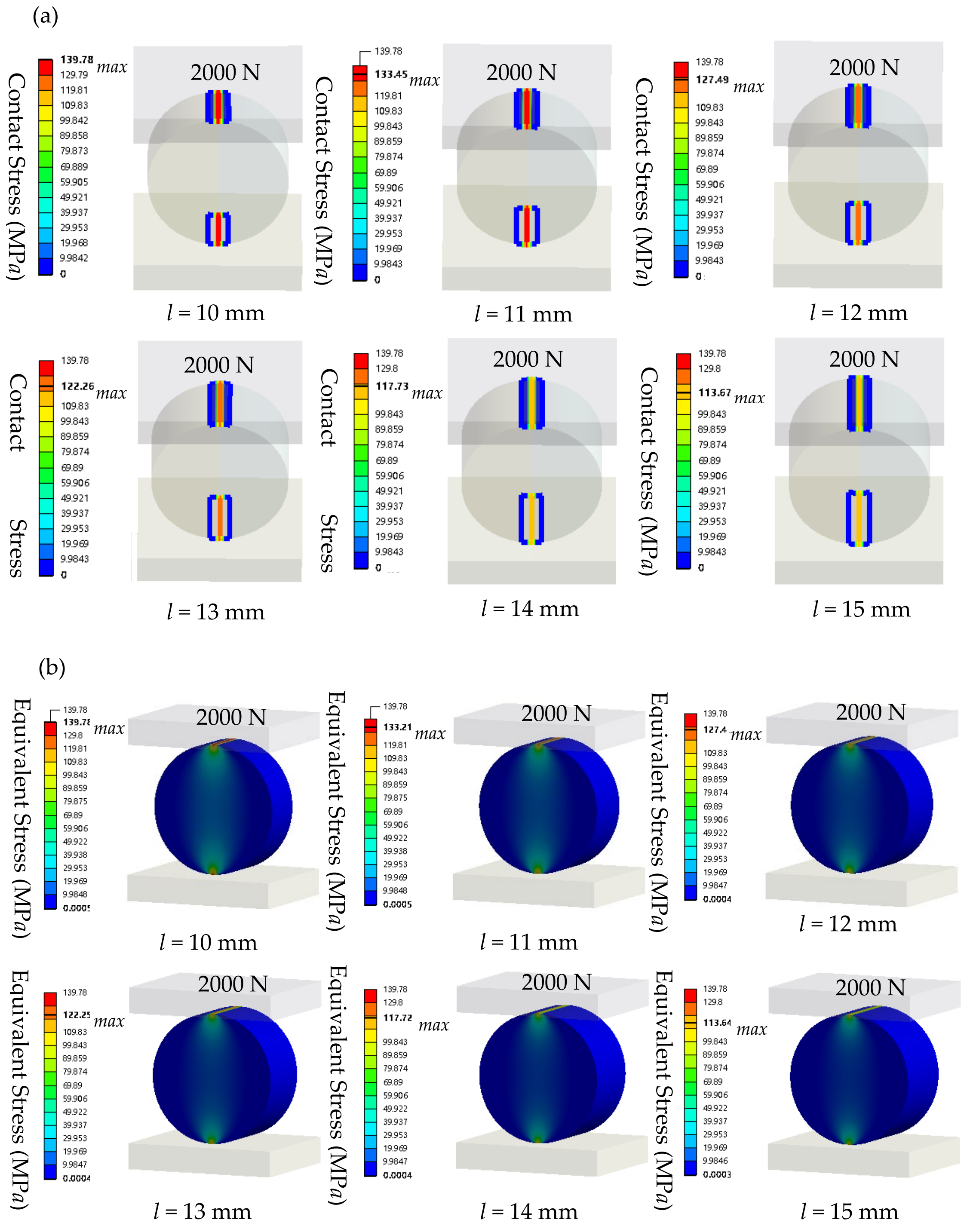

Figure S3. Finite element simulated stress contours of the ML cylinders of different sizes under a normal load of 2000 N. a) Contact stress contours obtained for the ML cylinders with a radius of 12.5 mm and lengths ranging from 10 to 15 mm. b) Equivalent stress distribution contours.

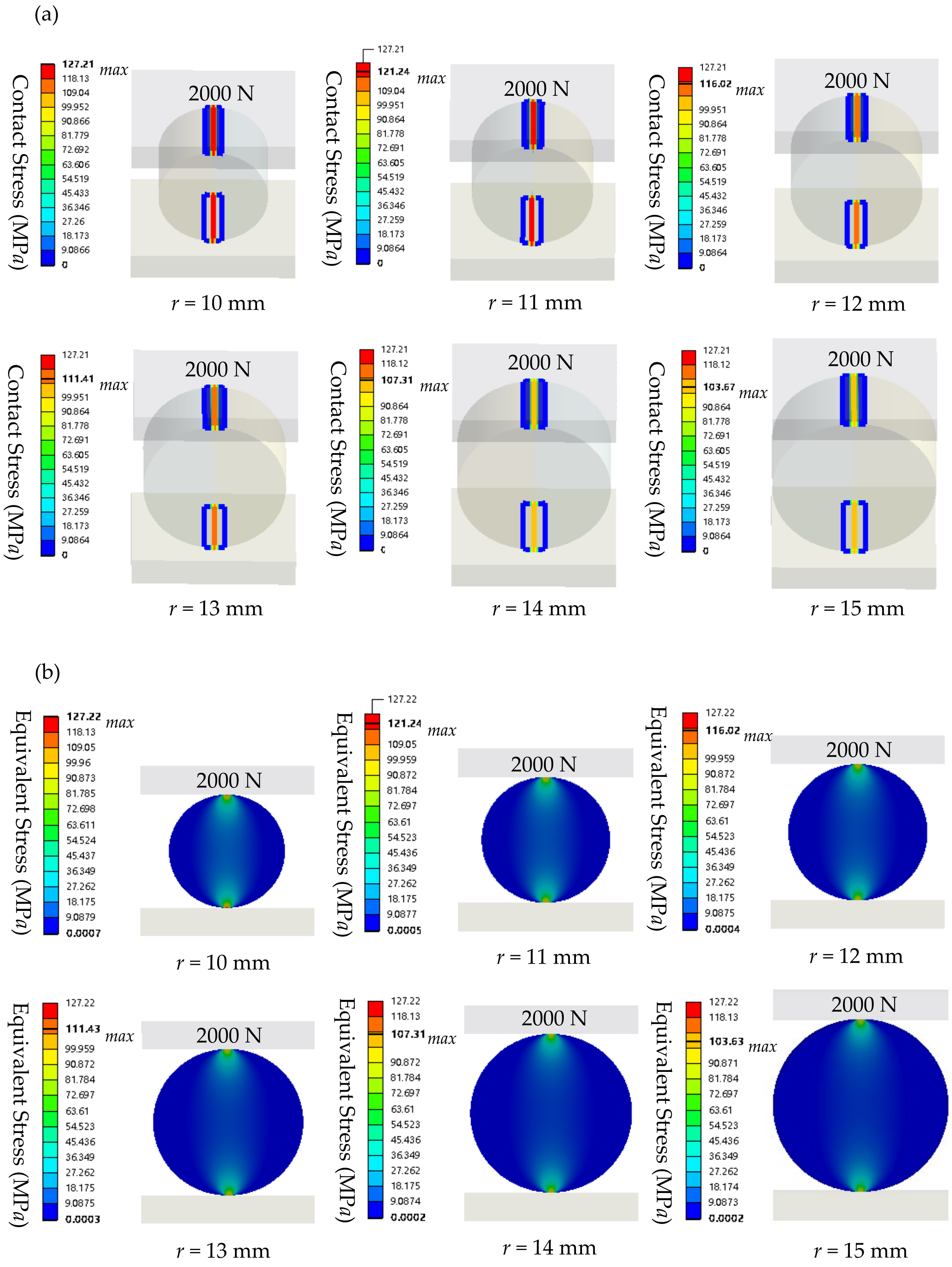

Figure S4. Finite element simulated Stress contours of the ML cylinders of different sizes under a normal load of 2000 N. a) Contact stress distribution contours obtained for the ML cylinders with a length of 15 mm and radius ranging from 10 to 15 mm. b) Equivalent stress distribution contours.

Table S1. Loading times required for the ML cylinders with different loading paths.

Normal Load *F*	Contact Half-Width mm	Normal Deformationm m	Loading Time at a Crosshead Speed of 10 mm/min	Loading Time at a Crosshead Speed of 5 mm/min	Loading Time at a Crosshead speed of 3 mm/min	Loading Time at a Crosshead Speed of 2 mm/min	Loading Time at a Crosshead Speed of 1 mm/min
500	0.353	0.020	0.118	0.235	0.392	0.588	1.177
1000	0.500	0.036	0.217	0.434	0.723	1.085	2.170
1500	0.612	0.052	0.309	0.619	1.032	1.547	3.095
2000	0.707	0.066	0.397	0.795	1.325	1.987	3.974
2500	0.790	0.080	0.482	0.964	1.607	2.410	4.820
3000	0.865	0.094	0.564	1.128	1.880	2.820	5.640
